# Advancing Public Health on the Changing Global Trade and Investment Agenda: Comment on "The Trans-Pacific Partnership: Is It Everything We Feared for Health?"

**DOI:** 10.15171/ijhpm.2016.129

**Published:** 2016-09-28

**Authors:** Anne Marie Thow, Deborah Gleeson

**Affiliations:** ^1^Menzies Centre for Health Policy, University of Sydney, Sydney, NSW, Australia.; ^2^School of Psychology and Public Health, La Trobe University, Melbourne, VIC, Australia.

**Keywords:** International Trade Agreements, Health, Policy Coherence, Policy Space

## Abstract

Concerns regarding the Trans-Pacific Partnership (TPP) have raised awareness about the negative public health impacts of trade and investment agreements. In the past decade, we have learned much about the implications of trade agreements for public health: reduced equity in access to health services; increased flows of unhealthy commodities; limits on access to medicines; and constrained policy space for health. Getting health on the trade agenda continues to prove challenging, despite some progress in moving towards policy coherence. Recent changes in trade and investment agendas highlight an opportunity for public health researchers and practitioners to engage in highly politicized debates about how future economic policy can protect and support equitable public health outcomes. To fulfil this opportunity, public health attention now needs to turn to strengthening policy coherence between trade and health, and identifying how solutions can be implemented. Key strategies include research agendas that address politics and power, and capacity building for both trade and health officials.

## Introduction

Agreement on the Trans-Pacific Partnership (TTP) in 2015 sent ripples of concern through the public health community. During negotiations stretching over five and a half years, public health advocates and researchers mounted a sustained campaign to identify potential concerns for health through analyses of leaked text and interaction with negotiators. Despite this concerted effort, the final text contains various provisions with negative implications for public health.

The paper by Ronald Labonté and colleagues in the July issue of the *International Journal of Health Policy and Management* presents a summary analysis of the potential impact on health of the world’s largest trade and investment agreement, the TTP Agreement.^1^ A strength of this study is its use of the recently published text of the TPP to identify the implications of specific provisions for health and health policy-making. This analysis identified notable public health ‘wins’ evident in the TPP text. However, it also supports the conclusions of previous research: international economic policy agreements can have a significant negative impact on health.

## What We Know About the Problem: Trade-Health Policy Incoherence

In the past decade, we have learned much about the implications of trade agreements for public health. There is potential for trade policy to contribute to equitable economic growth, in certain circumstances. In practice, however, trade and investment liberalization over the past six decades has coincided with ‘highly unequal patterns of income and wealth distribution.’^2^ There has also been increasing recognition of incoherence between outcomes of trade and investment liberalization, and goals of health policy.^3^ Policy coherence refers to the ‘systematic application of mutually reinforcing policies and integration of development concerns across government departments to achieve development goals along with national policy objectives.’^4^ The Sustainable Development Goals have prioritised policy coherence at the national and international level in Goal 17, following on from the Addis Ababa Accord.^5^

Quantitative studies and targeted health impact assessments have identified specifically how trade and investment agreements can reduce equity in access to health services, increase flows of commodities of public health concern (such as tobacco and unhealthy foods) and reduce access to medicines.^1,6-11^ Policy analyses of trade and investment agreement texts have highlighted the potential for specific provisions to limit access to medicines^12,13^ and constrain policy space for health, which refers to the scope that governments have to pursue policy priorities.^14-16^ Economic studies have also demonstrated the increased costs associated with provisions that expand and prolong monopolies on medicines and delay the availability of affordable generics.^17,18^ Case studies have illustrated the threat of investor-state dispute settlement (ISDS) mechanisms that enable corporations to claim compensation from governments in certain situations where their investments have been negatively affected by government action.^19,20^

The recognition that trade agreements need to allow policy space for governments to achieve other policy objectives, including public health, has also proved challenging to translate into practice. Most trade agreements incorporate the general exception in Article XX of the General Agreement on Tariffs and Trade for measures ‘necessary to protect human, animal or plant life or health,’^21^ which is intended to protect the right to regulate in the public interest, including for health purposes. However, the interpretation of this exception is a contested issue and its application is determined by arbitrators in the event of a dispute.

Trade policy-making takes place in an environment where industry voices are prominent and where public health concerns tend to be marginalised. In many cases, trade policy is influenced by industry to achieve objectives related to profit, with little consideration of implications for public health.^22^ Powerful lobby groups representing the economic interests of pharmaceutical, health services, agriculture, food, tobacco, and alcohol industries are seeking to strategically influence negotiations of trade and investment agreements.^23-26^ Indeed, provisions in recently signed agreements also explicitly provide for industry involvement in domestic policy-making.^1,14^

## Progress Towards Coherence

Despite these ongoing challenges, there has been some progress in moving towards policy coherence. One example is the exemption or ‘carve-out’ of tobacco control measures from ISDS in the TPP Agreement (Article 29.5). This means that for countries that elect to employ the exemption, the tobacco industry will not be able to use ISDS to seek compensation for tobacco control measures, in the manner in which tobacco company Phillip Morris has challenged tobacco plain packaging in Australia and large health warnings in Uruguay.^27^ However, even this safeguard is limited: it does not apply to the whole TPP text but is restricted to ISDS, meaning that tobacco companies may still be able to persuade states to pursue disputes on their behalf. Of even greater concern is that there are no exemptions for policy measures to address other public health issues such as the sale, marketing and labelling of alcohol and processed foods.^27^

Another example is the resistance shown by the non-US TPP countries to the US agenda to extend and expand monopolies on new medicines. Leaks of successive drafts of the TPP intellectual property chapter raised alarm amongst health and development organisations, resulting in considerable public pressure on governments to ensure that medicines remained affordable.^28^ As a result, some of the original US proposals were excluded from the TPP, and others were mitigated to a significant degree.^29,30^ However, provisions included in the final text of the TPP will still have a significant impact on access to medicines in developing countries, which were successful in securing only short and inflexible transition periods for implementation.^1^

## New Opportunities to Engage With a Changing Trade and Investment Agenda

A challenge for public health researchers is the need to engage with highly politicized and evolving economic agendas. The trade and investment agenda itself is changing. Multilateral negotiations at the World Trade Organization (WTO) have continued to stall, and there is substantial disagreement among major players about whether the WTO’s agenda should continue to focus on development, or on ‘new approaches’ for ‘meaningful outcomes’ in trade negotiations.^31^ New large-scale regional agreements, such as the TPP and Trans-Atlantic Trade and Investment Partnership (TTIP), have been celebrated as emerging from the multilateral vacuum to drive trade and investment liberalization forward.^32,33^ However, both of these agreements appear to have diminishing chances of ever entering into force.^34^

At the same time, the United Nations Conference on Trade and Development (UNCTAD) reports increasing dissatisfaction with ISDS processes, including a perception of bias towards investors among arbitrators of disputes.^35^ The Government of South Africa and Government of India have recently made the policy decision to terminate the majority of their bilateral investment treaties, in the interests of protecting policy space for domestic priorities. A range of model bilateral investment treaties at the national and regional level now contain very specifically circumscribed definitions of investment and the protections that will be offered to foreign investors, in an effort to expand domestic policy space.^2,35^ Countries are introducing a range of new approaches to preserve the right to regulate and ensure responsible development.^35^

Overall, these trends speak to a broader dissatisfaction with – and perhaps even destabilization of – the neoliberal regime that has dominated economic policy discourse, highlighted recently by a critique of neoliberalism from within the International Monetary Fund.^36^ With core beliefs of this regime being repeatedly challenged in the wake of the global financial crisis, some have identified disorganization in the global economic policy regime as representing an opportunity for new theories and discourses to shape policy directions.^37-39^

There is an opportunity now for public health researchers and practitioners to contribute to this discourse a vision for trade and investment policy that protects and supports equitable public health outcomes.^40^ This vision would encompass a strategic understanding of opportunities for coherence between health and economic policy, and concrete policy options to achieve both health and economic policy goals. For public health to speak effectively into these changing discourses will, however, require new directions in research and practice.

## What Are the Future Needs for Public Health Research and Practice?

### Politics and Power

Further nuanced analyses of the consequences of trade and investment policy for public health will continue to help in identifying specific provisions of concern. However, there is also a need to turn public health attention to how solutions can be implemented. One aspect of this is to continue to provide technical input to trade and investment policy-makers regarding specific policy options to protect and promote public health.

However, to effect change in the trade and investment agenda we must also address politics and power.^41,42^ One key issue is to understand the roles and avenues of influence of different stakeholders, including industry actors, and how embedded power relationships function to prevent (or support) change for public health (see, for example, Gleeson et al^23^). This will require research that examines how industry exerts power in economic policy arenas, and how diverse stakeholder interests can be managed. A second but equally important issue is the imbalance of economic and political power that often exists in trade negotiations between developed and developing countries; this requires insights from political science about the dynamics of negotiations and how structural power is exercised through networks and coalitions, as demonstrated in Peter Drahos’ analysis of negotiations over access to medicines at the WTO.^43^

Engaging with questions of power and influence will also be vital to identifying opportunities for real progress towards policy coherence. This will include research that examines economic policy-maker perceptions of the policy space available to them, constraints due to global commitments, and underlying priorities.^44^ Understanding these dynamics can help identify specific opportunities to change discourse and policy-making to advance public health interests. Analysing power and political dynamics at multiple levels – global, regional, and national – will enhance public health understanding of potential points of intervention that could bridge silos within policy-making and help to identify alternative paradigms, to promote policy coherence between economic priorities and health objectives.

### Capacity Building

Public health researchers and practitioners can play a valuable role in increasing capacity of governments to put public health issues on the trade agenda. However, this will require training of public health policy-makers and practitioners to ‘analyse political context and understand complexities, and to frame arguments and act effectively in the political arena’ – capacities that are often overlooked.^45^ Policy-oriented capacity building would improve the ability of public health policy-makers and practitioners to engage in two key ways. First, being better equipped to speak into trade policy negotiation and implementation to ensure that flexibilities, exceptions and new processes for dispute settlement are negotiated and implemented in ways that support positive public health outcomes. This will require not only technical knowledge, but also appropriate language and knowledge of avenues through which to communicate potential implications for public health.^46^ Second, supporting public health capacity to develop strong arguments to counter industry advocacy, that give more prominence to health concerns.^6,14,26^ This necessitates engagement with political and economic agendas, and can support the development of new discourses around achieving policy coherence between trade and health.

Efforts to increase public health capacity also need to address national implementation of trade agreements. There is scope at the ratification and implementation stage to mitigate potential impact on health inequalities.^47^ Strong regional support and capacity building for health can also establish norms or policy frameworks to provide a counterbalance to regional trade commitments.^46,48^

## Conclusion

The negative impacts of trade and investment agreements on public health outcomes and policy-making are becoming increasingly clear. Technical support provided by public health academics, advocates and practitioners has so far proved helpful in preventing and mitigating these effects, but alone is insufficient in ensuring protection for public health. To effect change, public health must turn attention to political and economic agendas that are heavily influenced by industry actors. These actors stand to gain a lot from provisions that may potentially have negative public health effects. Research into politics and power in trade agreements, and investment in capacity building should be key pillars of the next phase of public health research and practice regarding trade and investment policy.

## Ethical issues

Not applicable.

## Competing interests

DG and AMT have received funding from various non-government (not-for profit) organisations and governments to attend speaking engagements related to trade agreements and public health.

## Authors’ contributions

Both authors contributed equally to the writing of this paper.

## Authors’ affiliations

^1^Menzies Centre for Health Policy, University of Sydney, Sydney, NSW, Australia. ^2^School of Psychology and Public Health, La Trobe University, Melbourne, VIC, Australia.
